# Environmental DNA: From Promise to Practice

**DOI:** 10.1002/ece3.74041

**Published:** 2026-07-15

**Authors:** Jessica Rieder, Arley Muth, Christyn Bailey

**Affiliations:** ^1^ School of Fisheries, Aquaculture, and Aquatic Sciences, College of Agriculture Auburn University Auburn Alabama USA; ^2^ John Wiley & Sons Hoboken New Jersey USA; ^3^ John Wiley & Sons Oxford UK

Environmental DNA (eDNA), the genetic material that organisms shed into water, soil, sediment, or air, has become a central tool in modern ecology and conservation science, reshaping how ecologists survey biodiversity and assess ecosystem health. Over the past two decades, researchers have used eDNA to characterize biological diversity (Altermatt et al. 2020; van der Heyde et al. 2020; Sullivan et al. 2025), assess the ecological status of ecosystems (Wang, Wang, Ai, et al. 2025), inform conservation planning (Mauvisseau et al. 2020), and monitor rare, endangered, or threatened (Wood et al. 2020; Khan et al. 2026; Moreno et al. 2026) and invasive (Lutz et al. 2020; Abbott et al. 2021; Giovacchini et al. 2026) species. The work now reaches across most of biodiversity research, from microbes (Almeida et al. 2021; Grbin et al. 2023) to vertebrates (Rusch et al. 2020; Milián‐García et al. 2026), across terrestrial (Dyson et al. 2024; Newton et al. 2025) and aquatic environments (Stat et al. 2017; Rossouw et al. 2025; Morissette et al. 2026), and across a growing range of sample types (Mariani et al. 2019; Banerjee et al. 2025; Zinger et al. 2025).

Such reach, however, is a recent departure from how biodiversity has long been measured. Biodiversity has long been measured with methods such as kick nets, trawling, and capture‐recapture (Twagirayezu et al. 2026). These approaches are labor‐intensive and time‐consuming and, most critically, cannot be scaled or carried out quickly enough to keep pace with accelerating biodiversity loss and environmental change. These methods have not lost their value; they remain informative and often work alongside eDNA (Cereghetti et al. 2026). Even so, against this backdrop and amid rising policy demands, the need for more efficient tools is pressing, and the maturation of eDNA methods now places the field at a critical juncture between innovation and implementation (Sepulveda et al. 2020; Strand et al. 2020).

Reaching this point took around two decades of conceptual and methodological development. The groundwork for non‐microbial eDNA was laid by Willerslev and colleagues, who were the first to show that plant and animal DNA could be recovered directly from permafrost and temperate sediments (Willerslev et al. 2003), establishing that environmental samples retain identifiable genetic traces of the organisms present. The modern eDNA era, however, is most often credited to Ficetola et al. (2008), who assessed invasive bullfrog distribution from water samples and demonstrated that target DNA could be detected directly from the environment. The field then expanded from target‐species detection toward communities and management tools: Jerde et al. (2011) applied “sight‐unseen” detection to invasive carp, showing that eDNA could inform biosecurity before a species is ever observed; Taberlet et al. (2012) consolidated the conceptual and terminological framework around “environmental DNA”; and Thomsen et al. (2012) showed that metabarcoding of seawater could recover whole marine fish communities, shifting the goal from detecting a single target species to characterizing entire assemblages. A major milestone arrived in 2015, when Miya et al. (2015) developed the MiFish “universal” metabarcoding primers targeting the hypervariable region of the 12S rRNA gene, which has become the de facto marker for fish metabarcoding.

Building on these milestones, eDNA research has grown rapidly since the mid‐2010s, propelled by parallel advances in field and laboratory protocols, sequencing technology, and bioinformatics. On the field and laboratory side, systematic comparisons of capture, filtration, preservation, and extraction methods clarified which choices most affect detection sensitivity and DNA yield, moving the community toward optimized and repeatable protocols (Deiner et al. 2015; Eichmiller et al. 2016; Lear et al. 2018). In sequencing, the shift to high‐throughput platforms made community‐scale metabarcoding feasible, while the subsequent arrival of portable, real‐time long‐read devices such as Oxford Nanopore's MinION brought sequencing capacity into the field and into lower‐resourced settings (Garlapati et al. 2019). Alongside these, dedicated bioinformatic tools matured: metabarcoding‐specific suites such as OBITools (Boyer et al. 2016) streamlined read processing and taxonomic assignment, while denoising algorithms like DADA2 (Callahan et al. 2016), together with integrated platforms such as QIIME 2 (Bolyen et al. 2019), enabled the resolution of exact amplicon sequence variants rather than coarser operational taxonomic units.

These gains expanded what eDNA could survey, across both space and time. Deiner et al. (2016) showed that metabarcoding of river water could integrate biodiversity across an entire catchment, recovering hundreds of eukaryotic families spanning aquatic and terrestrial taxa and establishing freshwater eDNA as a landscape‐scale survey tool. Less than a decade later, building on Clare et al. (2022) and Lynggaard et al. (2022) filtered air at the Copenhagen Zoo and, through metabarcoding of airborne eDNA, detected dozens of vertebrate species, including mammals, birds, fish, one amphibian, and one reptile, opening terrestrial vertebrate monitoring quite literally from thin air. Kjær et al. (2022) also pushed eDNA's temporal limits, recovering genetic material from Greenland sediments at least two million years old and demonstrating the method's power to reconstruct the ecology and evolution of ancient communities. Together, these advances cemented eDNA as a reliable monitoring tool and began its shift from a method for observing biodiversity to one for actively managing it.

More recently, attention has shifted toward operational practice and management applications (Sepulveda et al. 2020). These efforts include embedding eDNA in regulatory bioassessment (e.g., the EU Water Framework Directive; Hering et al. 2018), standardizing protocols (e.g., the Molecular Lab Network; U.S. Department of the Interior 2019), and advancing emerging approaches such as quantitative eDNA (Smith et al. 2026; Rourke et al. 2026), environmental RNA (Benedicenti et al. 2024; Zou et al. 2025; Lopez et al. 2026), and machine learning (Koh 2026).

This Thematic Collection, jointly curated by *Aquaculture, Fish and Fisheries* and *Ecology and Evolution*, sets out to illustrate how environmental DNA has evolved into an integrative framework for biodiversity research, linking taxa, ecosystems, and regions across scales. Across this collection, studies document biodiversity from microbial communities in mangrove forests in Indonesia and lakes in New Zealand and Italy to diverse freshwater and marine fishes in major river basins and coastal systems in China, Taiwan, and Switzerland. Higher vertebrates are examined across distinct biogeographic contexts, from Rice's whale (*Balaenoptera ricei*) in North American waters to birds such as the great tit ( *Parus major* ) in Europe and pollinators including the African honey bee ( *Apis mellifera scutellata* ) in Kenya, alongside experimental and applied studies on zebrafish ( *Danio rerio* ), sea lamprey ( *Petromyzon marinus* ), and invertebrate assemblages in both tropical and temperate regions and across a variety of sample types (Figure 1).

**FIGURE 1:**
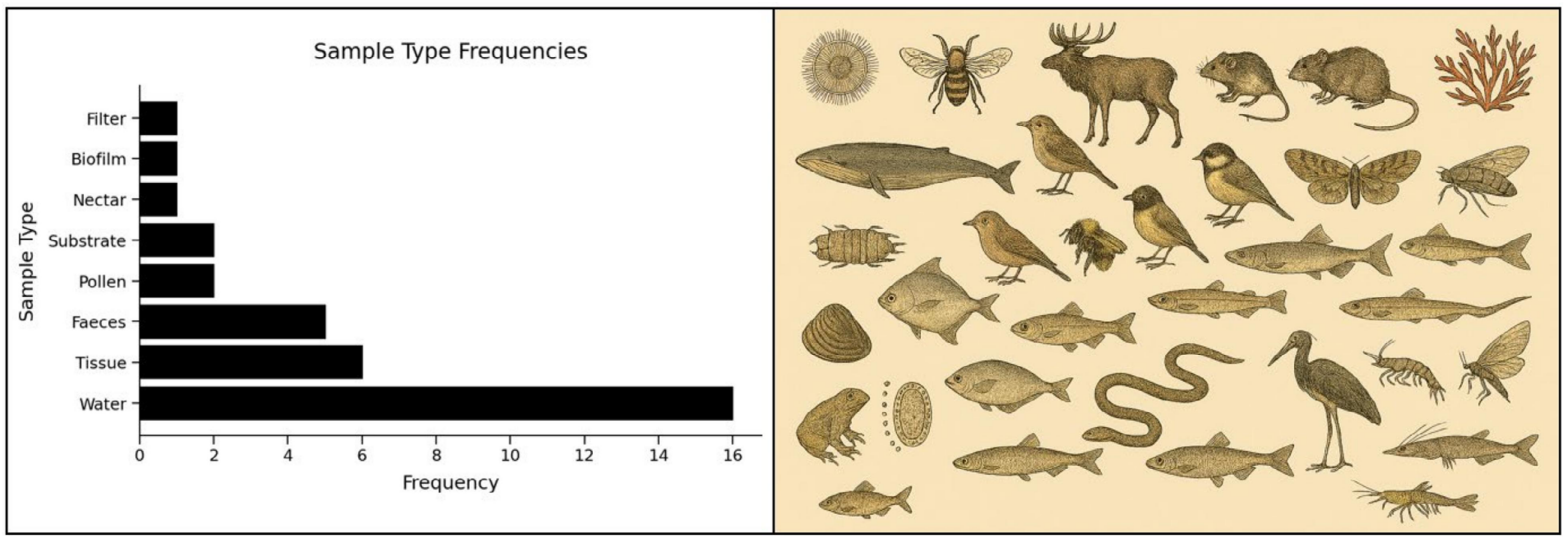
Overview of the sample types and taxonomic groups represented across the thematic collection. (Left) Frequency of each sample type used among the contributing studies, shown as a horizontal bar graph. (Right) Representative illustrations of the taxa investigated, spanning aquatic, terrestrial, and microbial systems and including primary producers, invertebrates, fishes, other aquatic fauna, amphibians and reptiles, birds, mammals, and microbes. The figure is a general summary and does not depict every individual study.

This thematic collection brings together contributions that reflect the breadth and momentum of the field, while underscoring the need for integrated perspectives on its continued development. These studies trace a progression across four interconnected fronts: methodological innovation; the widening of biodiversity monitoring and conservation across taxa and habitats; ecological and trophic insight into how species feed and interact; and the translation of detection into applied management, biosurveillance, and emerging frontiers that highlight both the challenges and the future opportunities of eDNA research. Taken together, these contributions demonstrate that eDNA is no longer simply an emerging technique but an increasingly influential approach for understanding and managing biological systems in a rapidly changing world. Notably, the studies draw on expertise from ecology, molecular biology, bioinformatics, fisheries science, and conservation practice, reflecting the inherently interdisciplinary nature of the field.

The first group sharpens the molecular and analytical toolkit. O'Kane et al. (2025) designed and validated eight blocking primers that suppress sea‐lamprey reads by more than 99.9%, allowing a single universal 12S marker to recover a taxonomically broad suite of host species in place of multiple taxon‐specific assays. Targeted detection advanced through new species‐specific and multiplex assays: an *rbcL* qPCR for a non‐indigenous Hawaiian macroalga *Acanthophora spicifera* (Nichols et al. 2025); a duplex digital PCR for two invasive Florida fishes, bullseye snakehead and Asian swamp eel (Bloch et al. 2026); and a multiplex droplet digital PCR that detects the morphologically identical larvae of quagga ( *Dreissena bugensis* ) and zebra mussels ( *Dreissena polymorpha* ), yielding first records of quagga mussels in Lakes Annecy and Aiguebelette (Vautier and Domaizon 2026).

Other research directly addressed accuracy and bias in metabarcoding studies. Sadyrova et al. (2025) evaluated two ITS2 primer sets (ITS‐S2F/ITS4 and UniPlant F/R) and found that both underrepresented graminoids. Vallin et al. (2026) tested two plant markers (ITS2 and trnL) in controlled feeding trials with sheep, underscoring how strongly marker choice shapes dietary estimates. Schoenemann et al. (2025) compared ITS2 and *rbcL* markers for bumble‐bee (*Bombus*) queen pollen loads, with *rbcL* detecting roughly twice the pollen diversity. Finally, Hall et al. (2025) paired traditional microscopy with 18S and COI metabarcoding in plankton mesocosm studies, finding that microscopy quantified abundance more precisely while metabarcoding captured broader taxonomic diversity, reinforcing the view that eDNA complements rather than replaces conventional survey methods.

Reliable interpretation depends as much on reference data and reproducible workflows as on the assays themselves, and several contributions turned to these foundations. Mai and Li (2026) introduced a semi‐automated workflow, *PhyloRef*, to flag and remove anomalous reference sequences, while Zhou et al. (2025) assembled a curated barcode library for Yellow River fishes, revealing that earlier surveys had severely underestimated the basin's diversity. Larekeng et al. (2026) applied a reproducible 18S/QIIME 2 pipeline to mangrove microbial communities, showing how environmental gradients in light, salinity, and nutrient availability shape species richness. Jones‐Slobodian et al. (2025) asked whether discarded filters from a national water‐quality network could be repurposed for fish biomonitoring and found they could not; a negative result still sharpens protocol guidance. Drawing these threads together, Wu et al. (2026) synthesized 15 years of method development and argue for standardized guidelines spanning sampling, extraction, and bioinformatics to improve reproducibility and cross‐study comparability.

A second group of articles widens the range of organisms and environments to which eDNA can be applied. In aquatic systems, multiple studies used eDNA metabarcoding to reconstruct fish assemblages across scales and biomes. In a year‐long study of 24 offshore sites, Wang, Wang, Hanafi‐Portier, et al. (2025) detected 1026 marine fish taxa, advancing understanding of offshore fish biodiversity in Taiwan and underscoring the role of oceanographic processes in shaping these communities. Zhao et al. (2025) surveyed the Jinsha (upper Yangtze) River during the initial phases of the basin's 10‐year fishing ban, documenting 61 fish species, including four nationally protected and seven invasive species, and establishing a baseline against which the ban's effectiveness can be assessed in future surveys. Comparable surveys tracked change in other systems: community instability and a trend toward miniaturization in the Xijiang River (Ma et al. 2025), high‐elevation fish diversity in the Yarlung Zangbo on the Tibetan Plateau (Wang, Wu, Li, et al. 2025), and seasonal community structure in Erhai Lake (Shu et al. 2026). Encouragingly, a survey of the once heavily polluted Mersey Estuary (UK) documented a resurgence in fish diversity, with several species detected for the first time since the mid‐nineteenth century (Jackman et al. 2026). The same sensitivity resolves the cryptic and the unwanted, clarifying the status of an elusive loach in Switzerland (Dubey et al. 2026) and screening ballast water for incoming alien taxa (Li et al. 2025).

Beyond fish, contributions reach into microbial and terrestrial realms: microbial and diatom communities in lowland and high‐altitude lakes (Mauro et al. 2025; Rimet et al. 2025), captivity‐driven change in a toad's gut mycobiome (Bradshaw et al. 2026), and benthic‐invertebrate metacommunity processes in a free‐flowing river (Martini et al. 2026). A further set of studies expanded the source of the DNA itself, drawing on invertebrate‐ and specimen‐derived material rather than water: invertebrate‐derived DNA from the guts of blood‐feeding leeches recovered vertebrate hosts and yielded the detection of a rare litter frog (Irawan and Eguchi 2025); DNA barcoding incorporated neglected insect larvae into forest biodiversity inventories (Sire et al. 2025); and barcoding of collected specimens resolved the vertical stratification of tropical moth communities between the canopy and understory (Böttger et al. 2026).

A third group of articles used eDNA tools to explore trophic ecology and species interactions. Several studies applied dietary metabarcoding to generalist foragers. Coomes et al. (2025) documented spatio‐temporal and cohort variation in the diet of a model passerine, the great tit ( *Parus major* ), in which the winter moth ( *Operophtera brumata* ) ranked among the most prevalent prey, but not necessarily the most important. Chege et al. (2025) showed that forage use by the African honey bee ( *Apis mellifera scutellata* ) in Kenya shifted with season and landscape, spanning 224 plant species across 65 families, with non‐native species pollen dominating the nutrition.

Two further studies asked how similar species coexist. On the one hand, using fecal metabarcoding, Kanishka et al. (2025) found high dietary overlap between two co‐occurring Australian mammals, the common brushtail possum ( *Trichosurus vulpecula* ) and bush rat ( *Rattus fuscipes* ), yet fine‐scale partitioning was sufficient to permit coexistence. On the other hand, Wang, Yang, Zhang, et al. (2025) found broader niche differentiation among three terrestrial isopods ( *Armadillidium vulgare* , *Sphaeroma raffaelei*, and *Trachelipus semiproiectus* ). Together, these studies highlight the importance of dietary specialization and resource partitioning, even at fine scales, in facilitating species coexistence.

The same approach was applied to vertebrate predators and their prey. On land, Acharya et al. (2026) combined fecal metabarcoding with microscopy to characterize the diet of tigers ( *Panthera tigris* ) in Nepal's Chitwan National Park, where spotted deer dominated and no livestock was detected, indicating the importance of protected wildlife areas. Yang et al. (2026) mapped the fish prey supporting black storks ( *Ciconia nigra* ) across their key foraging wetlands, informing conservation of this protected piscivore. In aquatic systems, Wang et al. (2026) sequenced the gastric contents of three pomfret fishes ( *Pampus argenteus* , *P. punctatissimus* , and *P. cinereus* ), revealing pronounced inter‐specific dietary variation consistent with resource partitioning among these sympatric congeners. Silliman et al. (2026) used eDNA metabarcoding to characterize the fish prey community within the core feeding habitats of the critically endangered Rice's whale (*Balaenoptera ricei*), recovering 99 species across 62 families and exceeding the diversity captured by concurrent trawl surveys, thereby demonstrating the value of eDNA as a complementary survey tool while underscoring the continued need for reference‐library development. Finally, Fell et al. (2025) extended the logic from individual diets to whole interaction networks, showing how non‐native plants restructure bird‐plant frugivory networks in a human‐modified tropical landscape in Panama.

Finally, a group of studies pushed eDNA from observation toward management and surveillance, and toward the field's next methodological frontier. In aquaculture and invasive‐species management, Liang et al. (2026) related eDNA concentration to the biomass of farmed Pacific white shrimp ( *Litopenaeus vannamei* ), offering a non‐invasive route to monitoring stock density, while Collins et al. (2026) used quantitative eDNA to track the spread of non‐native tilapia, including the blue‐spotted tilapia ( *Oreochromis leucostictus* ), across Tanzanian aquaculture and natural waters. Dahlquist et al. (2025) showed that eDNA can complement rather than replace traditional microscopy in the surveillance of invasive dreissenid mussels (*Dreissena* spp.), improving early detection when the two methods are combined. For disease and biosecurity, Atalah et al. (2026) surveyed 287 lakes across Aotearoa New Zealand and recovered 412 putative bacterial pathogens, establishing eDNA as a broad‐spectrum screening tool, while Rieder et al. (2025) reviewed emerging tools for aquaculture pathogen detection, including miniaturized and portable field labs, CRISPR‐based assays, eRNA, and metatranscriptomics. Pointing furthest ahead, Lindsay et al. (2026) moved beyond presence/absence to environmental messenger RNA, detecting stressor‐responsive gene transcripts in zebrafish ( *Danio rerio* ) exposed to a chemical contaminant and raising the prospect that environmental nucleic acids may report not only which organisms are present but how they are faring.

The contributions in this collection highlight the trajectory the field's own history predicts: from demonstrating that eDNA works, to making it work reliably, to deploying it where biodiversity is monitored, managed, and conserved. Where eDNA goes next is already visible at the edges of this collection. The most immediate frontier is the shift from presence to quantity and function. Beyond cataloging which taxa are present, eDNA is increasingly used to estimate abundance and biomass (Liang et al. 2026; Vautier and Domaizon 2026) and, through environmental RNA and messenger RNA, to infer how organisms are responding to their environment (Lindsay et al. 2026). Portable, real‐time sequencing and field‐deployable assays are moving analysis out of the laboratory and into lower‐resourced settings, while CRISPR‐based detection and metatranscriptomics extend the questions eDNA can address from identity to physiology and disease (Rieder et al. 2025). In parallel, integration across genetic markers, between molecular and traditional surveys, and with data streams such as remote sensing and machine learning, is poised to recast eDNA from a stand‐alone method into one layer of a richer, multi‐source picture of biodiversity (Wu et al. 2026).

These opportunities come with familiar constraints. Every eDNA inference still rests on the completeness of reference databases and the comparability of protocols, and several contributions here are candid that gaps in both remain rate‐limiting (Zhou et al. 2025; Mai and Li 2026; Wu et al. 2026). Quantitative interpretation, detection biases, and false positives and negatives demand continued validation against independent benchmarks, and conventional surveys remain essential partners rather than relics (Dahlquist et al. 2025; Hall et al. 2025). Realizing eDNA's promise at scale will therefore depend less on any single technical breakthrough than on standardization, transparent bioinformatics, and sustained collaboration among ecologists, molecular biologists, bioinformaticians, and the managers and policymakers who ultimately put the data to use. If the past two decades carried eDNA from proof of concept to practical tool, the next will be defined by how responsibly and how widely that tool is applied, turning a method that reveals what lives in an environment into one that helps decide how those environments, and the species that inhabit them, are protected.

## Author Contributions

**Jessica Rieder:** conceptualization (equal), writing – original draft (lead), writing – review and editing (lead). **Arley Muth:** conceptualization (equal), writing – original draft (supporting), writing – review and editing (supporting). **Christyn Bailey:** conceptualization (equal), writing – original draft (supporting), writing – review and editing (supporting).

## Funding

The authors have nothing to report.

## Conflicts of Interest

The authors declare no conflicts of interest.

## Data Availability

The authors have nothing to report.

## References

[ece374041-bib-0001] Abbott, C. , M. Coulson , N. Gagné , et al. 2021. “Guidance on the Use of Targeted Environmental DNA (eDNA) Analysis for the Management of Aquatic Invasive Species and Species at Risk.”

[ece374041-bib-0002] Acharya, H. B. , L. D. Bertola , D. Neupane , et al. 2026. “Diet and Prey Preference of Tigers ( *Panthera tigris* ) in and Around Chitwan National Park, Nepal.” Ecology and Evolution 16, no. 4: e73409. 10.1002/ece3.73409. 41970334 PMC13061741

[ece374041-bib-0003] Almeida, D. B. , C. Magalhães , Z. Sousa , et al. 2021. “Microbial Community Dynamics in a Hatchery Recirculating Aquaculture System (RAS) of Sole ( *Solea senegalensis* ).” Aquaculture 539: 736592. 10.1016/j.aquaculture.2021.736592.

[ece374041-bib-0004] Altermatt, F. , C. J. Little , E. Mächler , S. Wang , X. Zhang , and R. C. Blackman . 2020. “Uncovering the Complete Biodiversity Structure in Spatial Networks: The Example of Riverine Systems.” Oikos 129: oik.06806. 10.1111/oik.06806.

[ece374041-bib-0005] Atalah, J. , O. Laroche , J. K. Pearman , et al. 2026. “Uncovering Putative Bacterial Pathogens in Lakes in Aotearoa New Zealand Using Environmental DNA.” Ecology and Evolution 16, no. 2: e72818. 10.1002/ece3.72818. 41684828 PMC12892091

[ece374041-bib-0006] Banerjee, P. , J. P. Maity , N. Chatterjee , et al. 2025. “Harnessing Environmental DNA to Explore Frugivorous Interactions: A Case Study in Papaya ( *Carica papaya* ) and Pineapple ( *Ananas comosus* ).” Environmental DNA 7, no. 5: e70196. 10.1002/edn3.70196.

[ece374041-bib-0007] Benedicenti, O. , M. M. Amundsen , S. N. Mohammad , et al. 2024. “A Refinement to eRNA and eDNA‐Based Detection Methods for Reliable and Cost‐Efficient Screening of Pathogens in Atlantic Salmon Aquaculture.” PLoS One 19, no. 10: e0312337. 10.1371/journal.pone.0312337. 39432531 PMC11493300

[ece374041-bib-0008] Bloch, M. , E. Suarez , M. A. Miller , et al. 2026. “Development of a Duplex Digital PCR and Validation on eDNA Water Samples for Monitoring of the Asian Swamp Eel ( *Monopterus albus* /Javanensis) and Bullseye Snakehead (Channa Aurolineata/Marulius) in Florida, USA, Freshwater Ecosystems.” Ecology and Evolution 16, no. 2: e73088. 10.1002/ece3.73088. 41716589 PMC12912945

[ece374041-bib-0009] Bolyen, E. , J. R. Rideout , M. R. Dillon , et al. 2019. “Reproducible, Interactive, Scalable and Extensible Microbiome Data Science Using QIIME 2.” Nature Biotechnology 37, no. 8: 852–857. 10.1038/s41587-019-0209-9. PMC7015180 31341288

[ece374041-bib-0010] Böttger, D. , U. M. Diniz , A. Keller , S. D. Leonhardt , and G. Brehm . 2026. “Moth Communities Are More Diverse in the Understory Than in the Canopy of a Tropical Lowland Rainforest in NW Ecuador.” Ecology and Evolution 16, no. 4: e73337. 10.1002/ece3.73337.

[ece374041-bib-0011] Boyer, F. , C. Mercier , A. Bonin , Y. Le Bras , P. Taberlet , and E. Coissac . 2016. “Obitools: A Unix‐Inspired Software Package for DNA Metabarcoding.” Molecular Ecology Resources 16, no. 1: 176–182. 10.1111/1755-0998.12428. 25959493

[ece374041-bib-0012] Bradshaw, A. J. , S. Poo , T. E. Malter , et al. 2026. “Characterization of a Core Fungal Community and Captivity‐Induced Gut “Mycobiome” Change in Fowler's Toad ( *Anaxyrus fowleri* ).” Ecology and Evolution 16, no. 4: e73430. 10.1002/ece3.73430. 41970365 PMC13061749

[ece374041-bib-0013] Callahan, B. J. , P. J. McMurdie , M. J. Rosen , A. W. Han , A. J. A. Johnson , and S. P. Holmes . 2016. “DADA2: High‐Resolution Sample Inference From Illumina Amplicon Data.” Nature Methods 13, no. 7: 581–583. 10.1038/nmeth.3869. 27214047 PMC4927377

[ece374041-bib-0014] Cereghetti, E. , F. Altermatt , and L. Carraro . 2026. “Improving Species Abundance Information From River eDNA Metabarcoding Data.” Environmental DNA 8, no. 1: e70250. 10.1002/edn3.70250.

[ece374041-bib-0016] Chege, M. , M. B. Wambua , W. J. Kilonzo , S. Subramanian , and B. T. Nganso . 2025. “Seasonal and Landscape‐Driven Variations in Forage Resources of *Apis mellifera scutellata* : Implications for Pollination Sustainability and Colony Health in Taita Taveta County, Kenya.” Ecology and Evolution 15, no. 7: e71613. 10.1002/ece3.71613. 40584654 PMC12202974

[ece374041-bib-0017] Clare, E. L. , C. K. Economou , F. J. Bennett , et al. 2022. “Measuring Biodiversity From DNA in the Air.” Current Biology 32, no. 3: 693–700.e5. 10.1016/j.cub.2021.11.064. 34995488

[ece374041-bib-0018] Collins, R. A. , A. D. Saxon , A. H. Shechonge , M. A. Kishe , B. P. Ngatunga , and M. J. Genner . 2026. “Environmental DNA‐Based Quantification of an Invasive Tilapia Species in Tanzanian Inland Aquaculture.” Aquaculture, Fish and Fisheries 6, no. 2: e70237. 10.1002/aff2.70237.

[ece374041-bib-0019] Coomes, J. R. , J. P. Cuff , M. S. Reichert , G. L. Davidson , W. O. C. Symondson , and J. L. Quinn . 2025. “Spatio‐Temporal Variation in Diet Among Age and Sex Cohorts of a Model Generalist Bird Species, the Great Tit *Parus major* : New Insights Revealed by DNA Metabarcoding.” Ecology and Evolution 15, no. 7: e71565. 10.1002/ece3.71565. 40661920 PMC12256774

[ece374041-bib-0020] Dahlquist, Z. , D. L. Miller , S. J. Amish , L. Howard , M. McCartney , and G. Luikart . 2025. “Invasive Species Monitoring Is Improved by Combining eDNA qPCR and Traditional Microscopy Methods.” Aquaculture, Fish and Fisheries 5, no. 4: e70075. 10.1002/aff2.70075.

[ece374041-bib-0021] Deiner, K. , E. A. Fronhofer , E. Mächler , J.‐C. Walser , and F. Altermatt . 2016. “Environmental DNA Reveals That Rivers Are Conveyer Belts of Biodiversity Information.” Nature Communications 7, no. 1: 12544. 10.1038/ncomms12544. PMC5013555 27572523

[ece374041-bib-0022] Deiner, K. , J. C. Walser , E. Mächler , and F. Altermatt . 2015. “Choice of Capture and Extraction Methods Affect Detection of Freshwater Biodiversity From Environmental DNA.” Biological Conservation 183: 53–63. 10.1016/j.biocon.2014.11.018.

[ece374041-bib-0023] Dubey, S. , M. Pompini , and J. Golay . 2026. “Revisiting the Conservation Status of *Cobitis taenia* in Switzerland Using Environmental DNA.” Aquaculture, Fish and Fisheries 6, no. 1: e70186. 10.1002/aff2.70186.

[ece374041-bib-0024] Dyson, K. , A. P. Nicolau , K. Tenneson , et al. 2024. “Coupling Remote Sensing and eDNA to Monitor Environmental Impact: A Pilot to Quantify the Environmental Benefits of Sustainable Agriculture in the Brazilian Amazon.” PLoS One 19, no. 2: e0289437. 10.1371/journal.pone.0289437. 38354171 PMC10866516

[ece374041-bib-0025] Eichmiller, J. J. , L. M. Miller , and P. W. Sorensen . 2016. “Optimizing Techniques to Capture and Extract Environmental DNA for Detection and Quantification of Fish.” Molecular Ecology Resources 16, no. 1: 56–68. 10.1111/1755-0998.12421. 25919417

[ece374041-bib-0026] Fell, A. , C. Bello , A. B. Duthie , et al. 2025. “Non‐Native Plants Alter Bird‐Plant Frugivory Network Structure in a Human‐Modified Tropical Landscape.” Ecology and Evolution 15, no. 12: e72620. 10.1002/ece3.72620. 41377733 PMC12686967

[ece374041-bib-0027] Ficetola, G. F. , C. Miaud , F. Pompanon , and P. Taberlet . 2008. “Species Detection Using Environmental DNA From Water Samples.” Biology Letters 4, no. 4: 423–425. 10.1098/rsbl.2008.0118. 18400683 PMC2610135

[ece374041-bib-0028] Garlapati, D. , B. Charankumar , K. Ramu , P. Madeswaran , and M. V. Ramana Murthy . 2019. “A Review on the Applications and Recent Advances in Environmental DNA (eDNA) Metagenomics.” Reviews in Environmental Science and Bio/Technology 18, no. 3: 389–411. 10.1007/s11157-019-09501-4.

[ece374041-bib-0029] Giovacchini, S. , E. Mirone , A. Bruno , et al. 2026. “Conservation Planning and Reporting Implications of qPCR‐Based Multi‐Species eDNA Detection Under EU Environmental Regulations.” Environmental Management 76, no. 6: 211. 10.1007/s00267-026-02503-3. 42250110 PMC13242467

[ece374041-bib-0030] Grbin, D. , S. Geček , A. Miljanović , et al. 2023. “Comparison of Exoskeleton Microbial Communities of Co‐Occurring Native and Invasive Crayfish Species.” Journal of Invertebrate Pathology 201: 107996. 10.1016/j.jip.2023.107996. 37783231

[ece374041-bib-0031] Hall, C. A. M. , N. Henry , O. Canals , G. Consing , N. Rodríguez‐Ezpeleta , and A. M. Lewandowska . 2025. “DNA Metabarcoding as a Tool to Study Plankton Responses to Warming and Salinity Change in Mesocosms.” Ecology and Evolution 15, no. 9: e72125. 10.1002/ece3.72125. 40958841 PMC12434319

[ece374041-bib-0032] Hering, D. , A. Borja , J. I. Jones , et al. 2018. “Implementation Options for DNA‐Based Identification Into Ecological Status Assessment Under the European Water Framework Directive.” Water Research 138: 192–205. 10.1016/j.watres.2018.03.003. 29602086

[ece374041-bib-0033] Irawan, A. D. , and K. Eguchi . 2025. “Application of Invertebrate‐Derived DNA Barcoding (iDNA) in Blood Sucking Leeches From West Sumatra: A Discovery of Blue‐Eyed Litter Frog Leptobrachium Waysapuntiense.” Ecology and Evolution 15, no. 10: e72235. 10.1002/ece3.72235. 41041396 PMC12486191

[ece374041-bib-0034] Jackman, J. M. , N. G. Sales , C. Benvenuto , et al. 2026. “Multi‐Seasonal eDNA Metabarcoding Highlights a Resurgence in Fish Diversity Across a Severely Impacted Estuarine Ecosystem.” Aquaculture, Fish and Fisheries 6, no. 2: e70201. 10.1002/aff2.70201.

[ece374041-bib-0035] Jerde, C. L. , A. R. Mahon , W. L. Chadderton , and D. M. Lodge . 2011. ““Sight‐Unseen” Detection of Rare Aquatic Species Using Environmental DNA.” Conservation Letters 4, no. 2: 150–157. 10.1111/j.1755-263X.2010.00158.x.

[ece374041-bib-0036] Jones‐Slobodian, D. N. , D. Wieferich , N. Fierer , J. Craine , and A. Sepulveda . 2025. “Turning Trash Into Treasure: Leveraging Discarded Filters for National‐Scale Aquatic eDNA Biomonitoring.” Aquaculture, Fish and Fisheries 5, no. 4: e70104. 10.1002/aff2.70104.

[ece374041-bib-0037] Kanishka, A. M. , C. MacGregor , L. E. Neaves , et al. 2025. “Quantifying the Dietary Overlap of Two co‐Occurring Mammal Species Using DNA Metabarcoding to Assess Potential Competition.” Ecology and Evolution 15, no. 4: e71274. 10.1002/ece3.71274. 40225886 PMC11992362

[ece374041-bib-0038] Khan, A. A. , B. L. M. Keber , K. G. Harder , J. L. Ogden , and M. F. Docker . 2026. “Species‐Specific Environmental DNA (eDNA) Assay for Monitoring the Distribution of the Endangered Carmine Shiner *Notropis percobromus* in Manitoba.” Environmental Biology of Fishes 109, no. 2: 78. 10.1007/s10641-026-01848-2.

[ece374041-bib-0039] Kjær, K. H. , M. Winther Pedersen , B. De Sanctis , et al. 2022. “A 2‐Million‐Year‐Old Ecosystem in Greenland Uncovered by Environmental DNA.” Nature 612, no. 7939: 283–291. 10.1038/s41586-022-05453-y. 36477129 PMC9729109

[ece374041-bib-0040] Koh, J. C. O. 2026. “Explainable Multimodal Machine Learning Using Combined Environmental DNA and Biogeographic Features for Ecosystem Biomonitoring.” Environmental DNA 8, no. 1: e70232. 10.1002/edn3.70232.

[ece374041-bib-0041] Larekeng, S. H. , M. Basyuni , A. A. Aznawi , et al. 2026. “eDNA Metabarcoding Reveals Microbial Community Composition in Tropical Mangrove Forests in Makassar, Indonesia.” Ecology and Evolution 16, no. 1: e72740. 10.1002/ece3.72740. 41527541 PMC12790866

[ece374041-bib-0042] Lear, G. , I. Dickie , J. Banks , et al. 2018. “Methods for the Extraction, Storage, Amplification and Sequencing of DNA From Environmental Samples.” New Zealand Journal of Ecology 42, no. 1: 1–15. 10.20417/nzjecol.42.9.

[ece374041-bib-0043] Li, H. , H. Jia , J. Peng , X. Peng , Z. Ren , and H. Zhang . 2025. “Monitoring Alien Species Diversity in Ballast Water Based on Environmental DNA Metabarcoding.” Ecology and Evolution 15, no. 10: e72320. 10.1002/ece3.72320. 41098891 PMC12519623

[ece374041-bib-0044] Liang, G. , S. Wang , S. Wang , Y. Jiang , and Y. Qin . 2026. “Research on Quantitative Analysis Technology for Pacific White Shrimp Density Based on Environmental DNA.” Ecology and Evolution 16, no. 4: e73299. 10.1002/ece3.73299. 41993729 PMC13078953

[ece374041-bib-0045] Lindsay, D. L. , J. E. Mylroie , K. A. Gust , E. M. Cowan , and R. F. Lance . 2026. “Pattern of Detections Across Multiple Environmental Messenger RNAs (e‐mRNAs) in Stressor‐Exposed Zebrafish ( *Danio rerio* ).” Ecology and Evolution 16, no. 1: e72986. 10.1002/ece3.72986. 41583888 PMC12831017

[ece374041-bib-0046] Lopez, M. L. D. , A. Migneault , S. L. Trilesky , et al. 2026. “Design, Validation, and Implementation Considerations for Species‐Targeted Environmental RNA Assays to Detect Presence and Physiological State.” Environmental DNA 8, no. 2: e70272. 10.1002/edn3.70272.

[ece374041-bib-0047] Lutz, E. , P. E. Hirsch , K. Bussmann , et al. 2020. “Predation on Native Fish Eggs by Invasive Round Goby Revealed by Species‐Specific Gut Content DNA Analyses.” Aquatic Conservation: Marine and Freshwater Ecosystems 30, no. 8: 1566–1577. 10.1002/aqc.3409.

[ece374041-bib-0048] Lynggaard, C. , M. F. Bertelsen , C. V. Jensen , et al. 2022. “Airborne Environmental DNA for Terrestrial Vertebrate Community Monitoring.” Current Biology 32, no. 3: 701–707.e5. 10.1016/j.cub.2021.12.014. 34995490 PMC8837273

[ece374041-bib-0049] Ma, X. , F. Huang , R. Zhang , T. Liu , T. Zhang , and P. Zeng . 2025. “Environmental DNA Reveals the Fish Community Structure Exhibited Instability and Trend of Miniaturization in the Xijiang River Basin of the Guizhou.” Ecology and Evolution 15, no. 9: e71825. 10.1002/ece3.71825. 40978217 PMC12449034

[ece374041-bib-0050] Mai, Y. , and C. Li . 2026. “PhyloRef: A Semi‐Automated Workflow for eDNA Reference Database Curation via Phylogenetic Anomaly Detection.” Ecology and Evolution 16, no. 3: e73159. 10.1002/ece3.73159. 41766741 PMC12946455

[ece374041-bib-0051] Mariani, S. , C. Baillie , G. Colosimo , and A. Riesgo . 2019. “Sponges as Natural Environmental DNA Samplers.” Current Biology 29, no. 11: R401–R402. 10.1016/j.cub.2019.04.031. 31163139

[ece374041-bib-0052] Martini, J. , T. Fuß , M. V. Brasseur , et al. 2026. “Seasonal Metacommunity Processes of Benthic Invertebrates in the Vjosa/Aoos River.” Ecology and Evolution 16, no. 4: e73307. 10.1002/ece3.73307.

[ece374041-bib-0053] Mauro, M. , V. Villanova , M. L. Valvo , et al. 2025. “Environmental DNA: Preliminary Characterization of Microbiota in Three Sicilian Lakes.” Ecology and Evolution 15, no. 4: e71276. 10.1002/ece3.71276. 40290376 PMC12022232

[ece374041-bib-0054] Mauvisseau, Q. , E. Kalogianni , B. Zimmerman , M. Bulling , R. Brys , and M. Sweet . 2020. “eDNA‐Based Monitoring: Advancement in Management and Conservation of Critically Endangered Killifish Species.” Environmental DNA 2, no. 4: 601–613. 10.1002/edn3.92.

[ece374041-bib-0055] Milián‐García, Y. , P. Giroux , M. Docker , et al. 2026. “Community‐Based eDNA Metabarcoding for Monitoring Fish Biodiversity and Food Webs in the Peace‐Athabasca Delta.” PeerJ 14: e21341. 10.7717/peerj.21341. 42199828 PMC13200620

[ece374041-bib-0056] Miya, M. , Y. Sato , T. Fukunaga , et al. 2015. “MiFish, a Set of Universal PCR Primers for Metabarcoding Environmental DNA From Fishes: Detection of More Than 230 Subtropical Marine Species.” Royal Society Open Science 2, no. 7: 150088. 10.1098/rsos.150088. 26587265 PMC4632578

[ece374041-bib-0057] Moreno, D. , T. N. Kwan , B. Deagle , et al. 2026. “First eDNA Evidence for the Potential Functional Disappearance of the Endangered Maugean Skate Zearaja Maugeana in Bathurst Harbour, Australia.” Endangered Species Research 53: 215–224. 10.3354/esr01510.

[ece374041-bib-0058] Morissette, O. , G. Côté , M.‐A. Couillard , R. Pouliot , and L. Bernatchez . 2026. “Trait‐Based Biomonitoring Using eDNA Metabarcoding to Assess Anthropogenic Disturbances on Freshwater Fish Communities.” Molecular Ecology Resources 26, no. 3: e70131. 10.1111/1755-0998.70131. 41858251 PMC13003273

[ece374041-bib-0059] Newton, J. P. , M. E. Allentoft , P. W. Bateman , M. van der Heyde , and P. Nevill . 2025. “Targeting Terrestrial Vertebrates With eDNA: Trends, Perspectives, and Considerations for Sampling.” Environmental DNA 7, no. 1: e70056. 10.1002/edn3.70056.

[ece374041-bib-0060] Nichols, P. K. , A. R. Sherwood , K. M. S. Fraiola , et al. 2025. “Detection of a Non‐Indigenous Marine Macroalga ( *Acanthophora spicifera* ) With Environmental DNA From Surface Seawater.” Aquaculture, Fish and Fisheries 5, no. 6: e70135. 10.1002/aff2.70135.

[ece374041-bib-0061] O'Kane, C. , N. S. Johnson , K. T. Scribner , J. Kanefsky , W. Li , and J. D. Robinson . 2025. “Development of PCR Blocking Primers Enabling DNA Metabarcoding Analysis of Dietary Composition in Hematophagous Sea Lamprey.” Ecology and Evolution 15, no. 8: e71999. 10.1002/ece3.71999. 40852627 PMC12367867

[ece374041-bib-0062] Rieder, J. , A. Berezenko , A. Meziti , and I. Adrian‐Kalchhauser . 2025. “The Future of Pathogen Detection in Aquaculture: Miniature Labs, Field‐Compatible Assays, Environmental DNA and RNA, CRISPR and Metatranscriptomics.” Aquaculture, Fish and Fisheries 5, no. 3: e70062. 10.1002/aff2.70062.

[ece374041-bib-0063] Rimet, F. , C. Lemonnier , and B. Alric . 2025. “Stochasticity Shapes Microbial Communities in High‐Altitude Lakes, Whereas Species Selection and Homogenization Dispersal Are More Important in Lowland Lakes: Case of Benthic Diatoms in Alpine Lakes.” Ecology and Evolution 15, no. 9: e71977. 10.1002/ece3.71977. 40881665 PMC12389866

[ece374041-bib-0064] Rossouw, E. I. , S. von der Heyden , and N. Peer . 2025. “Aquatic eDNA Outperforms Sedimentary eDNA for the Detection of Estuarine Fish Communities in Subtropical Coastal Vegetated Ecosystems.” Journal of Fish Biology 107, no. 2: 520–534. 10.1111/jfb.70056. 40223581 PMC12360151

[ece374041-bib-0065] Rourke, M. L. , M. K. Broadhurst , A. M. Fowler , et al. 2026. “Correlating eDNA Indices of Relative Biomass and Abundance With Those Derived From Electrofishing for Two Ecologically Disparate Australian Freshwater Species.” Aquatic Sciences 88, no. 3: 90. 10.1007/s00027-026-01317-z.

[ece374041-bib-0066] Rusch, J. C. , M. Mojžišová , D. A. Strand , J. Svobodová , T. Vrålstad , and A. Petrusek . 2020. “Simultaneous Detection of Native and Invasive Crayfish and *Aphanomyces astaci* From Environmental DNA Samples in a Wide Range of Habitats in Central Europe.” NeoBiota 58: 1–32. 10.3897/neobiota.58.49358.

[ece374041-bib-0067] Sadyrova, M. , E. Martin , P. Ramsey , and L. Bullington . 2025. “Mock Plant Communities and a Large Mammal Case Study Reveal ITS2 Primer Bias Against Graminoids.” Ecology and Evolution 15, no. 9: e72102. 10.1002/ece3.72102. 40927319 PMC12414724

[ece374041-bib-0068] Schoenemann, K. , H. C. Lim , M. M. Keady , and D. E. Carr . 2025. “Pollen Foraging by Bumble Bee Queens During a Critical Nesting Period Revealed by DNA Metabarcoding.” Ecology and Evolution 15, no. 12: e72733. 10.1002/ece3.72733. 41438953 PMC12720142

[ece374041-bib-0069] Sepulveda, A. J. , N. M. Nelson , C. L. Jerde , and G. Luikart . 2020. “Are Environmental DNA Methods Ready for Aquatic Invasive Species Management?” Trends in Ecology & Evolution 35, no. 8: 668–678. 10.1016/j.tree.2020.03.011. 32371127

[ece374041-bib-0070] Shu, L. , A. Ludwig , H. Pan , et al. 2026. “Diversity and Spatiotemporal Distribution of Fish in a Highland Lake in China Based on Environmental DNA Metabarcoding.” Ecology and Evolution 16, no. 2: e73082. 10.1002/ece3.73082. 41684819 PMC12893789

[ece374041-bib-0071] Silliman, K. , L. A. Wilcox Talbot , M. Applegate , et al. 2026. “Evaluating Prey Availability for the Rice's Whale (*Balaenoptera ricei*) Based on Environmental DNA.” Ecology and Evolution 16, no. 1: e72789. 10.1002/ece3.72789. 41537137 PMC12796833

[ece374041-bib-0072] Sire, L. , C. Martin , G. Parmain , et al. 2025. “Incorporating Neglected Insect Larvae in Species Inventories: DNA Barcoding as an Effective Tool for All‐Stage Invertebrate Identification in Tree Holes.” Ecology and Evolution 15, no. 6: e71586. 10.1002/ece3.71586. 40567577 PMC12188026

[ece374041-bib-0073] Smith, I. S. , R. Bajno , E. Spice , et al. 2026. “High Replicability in Strong Correlation Between Goldfish Abundance, eDNA Detection Probability, and eDNA Concentration in Urban Ponds.” Environmental DNA 8, no. 2: e70259. 10.1002/edn3.70259.

[ece374041-bib-0074] Stat, M. , M. J. Huggett , R. Bernasconi , et al. 2017. “Ecosystem Biomonitoring With eDNA: Metabarcoding Across the Tree of Life in a Tropical Marine Environment.” Scientific Reports 7, no. 1: 12240. 10.1038/s41598-017-12501-5. 28947818 PMC5612959

[ece374041-bib-0075] Strand, D. A. , S. I. Johnsen , J. Rusch , T. Vrålstad , S. Ivar , and E. Brun . 2020. “The Surveillance Programme for *Aphanomyces astaci* in Norway 2020.”

[ece374041-bib-0076] Sullivan, A. R. , E. Karlsson , D. Svensson , et al. 2025. “Airborne eDNA Captures Three Decades of Ecosystem Biodiversity.” Nature Communications 16, no. 1: 11281. 10.1038/s41467-025-67676-7. PMC12717267 41413054

[ece374041-bib-0077] Taberlet, P. , E. Coissac , M. Hajibabaei , and L. H. Rieseberg . 2012. “Environmental DNA.” Molecular Ecology 21, no. 8: 1789–1793. 10.1111/j.1365-294X.2012.05542.x. 22486819

[ece374041-bib-0078] Thomsen, P. F. , J. Kielgast , L. L. Iversen , et al. 2012. “Monitoring Endangered Freshwater Biodiversity Using Environmental DNA.” Molecular Ecology 21, no. 11: 2565–2573. 10.1111/j.1365-294X.2011.05418.x. 22151771

[ece374041-bib-0080] Twagirayezu, E. , A. Kirkaldy , G. Ndatimana , et al. 2026. “Patterns and Adaptive Differences of Aquatic Macroinvertebrates in Protected and Non‐Protected Aquatic Systems of Rwanda.” Environmental Monitoring and Assessment 198, no. 5: 440. 10.1007/s10661-026-15313-1. 41963567

[ece374041-bib-0081] U.S. Department of the Interior . 2019. “Early Detection and Rapid Response.” https://www.doi.gov/invasivespecies/early‐detection‐and‐rapid‐response.

[ece374041-bib-0082] Vallin, H. , M. Fraser , R. J. Pakeman , and H. Hipperson . 2026. “Evaluating the Quantitative Accuracy and Application of DNA Metabarcoding for Dietary Reconstruction in Ruminants.” Ecology and Evolution 16, no. 1: e72878. 10.1002/ece3.72878. 41542380 PMC12801136

[ece374041-bib-0083] van der Heyde, M. , M. Bunce , G. Wardell‐Johnson , K. Fernandes , N. E. White , and P. Nevill . 2020. “Testing Multiple Substrates for Terrestrial Biodiversity Monitoring Using Environmental DNA Metabarcoding.” Molecular Ecology Resources 20, no. 3: 732–745. 10.1111/1755-0998.13148. 32065512

[ece374041-bib-0084] Vautier, M. , and I. Domaizon . 2026. “Advancing Environmental DNA Methods for Monitoring the Reproduction of Quagga and Zebra Mussels in Lakes.” Aquaculture, Fish and Fisheries 6, no. 3: e70252. 10.1002/aff2.70252.

[ece374041-bib-0085] Wang, H. , Q. Wu , S. Li , et al. 2025. “Environmental DNA Based Assessment of Fish Diversity in the Yarlung Zangbo River, Tibetan Plateau.” Ecology and Evolution 15, no. 11: e72496. 10.1002/ece3.72496. 41229699 PMC12602264

[ece374041-bib-0086] Wang, J. , Y. Yang , G. Zhang , W. Xu , and H. Liu . 2025. “Niche Differentiation of Three Terrestrial Isopod Species Based on DNA Metabarcoding.” Ecology and Evolution 15, no. 7: e71682. 10.1002/ece3.71682. 40584663 PMC12202779

[ece374041-bib-0087] Wang, W. , C. Liu , D. Lv , Y. Hu , G. Lv , and X. Shan . 2026. “High‐Throughput DNA Sequencing Reveals Gastric Content Composition and Inter‐Specific Variation in Pampus Fishes.” Ecology and Evolution 16, no. 3: e73123. 10.1002/ece3.73123. 41766735 PMC12946655

[ece374041-bib-0088] Wang, X. , X. Wang , S. Ai , et al. 2025. “Merging eDNA and Ecological Network Analysis to Assess Aquatic Ecosystem Status.” Ecological Indicators 176: 113727. 10.1016/j.ecolind.2025.113727.

[ece374041-bib-0089] Wang, Y.‐C. , D. Wang , M. Hanafi‐Portier , C.‐L. Wei , V. Denis , and W.‐J. Chen . 2025. “Environmental DNA Metabarcoding Reveals Distinct Spatial and Seasonal Patterns in Offshore Fish Communities in Eastern and Western Taiwan.” Aquaculture, Fish and Fisheries 5, no. 6: e70144. 10.1002/aff2.70144.

[ece374041-bib-0090] Willerslev, E. , A. J. Hansen , J. Binladen , et al. 2003. “Diverse Plant and Animal Genetic Records From Holocene and Pleistocene Sediments.” Science 300, no. 5620: 791–795. 10.1126/science.1084114. 12702808

[ece374041-bib-0091] Wood, S. A. , L. Biessy , J. L. Latchford , et al. 2020. “Release and Degradation of Environmental DNA and RNA in a Marine System.” Science of the Total Environment 704: 135314. 10.1016/j.scitotenv.2019.135314. 31780169

[ece374041-bib-0092] Wu, S. , Y. Wang , H. Qin , et al. 2026. “Environmental DNA (eDNA) Technology in Biodiversity and Ecosystem Health Research: Advances and Prospects.” Ecology and Evolution 16, no. 1: e72891. 10.1002/ece3.72891. 41522225 PMC12789655

[ece374041-bib-0093] Yang, X. , B. Liu , J. Wu , X. Yuan , D. Li , and S. Liu . 2026. “Utilizing Environmental DNA Metabarcoding to Assess Fish‐Based Food Resources in Key Foraging Areas of the Black Stork.” Ecology and Evolution 16, no. 4: e73526. 10.1002/ece3.73526. 42017118 PMC13092954

[ece374041-bib-0094] Zhao, Y. , Z. Wang , F. Hu , et al. 2025. “Fish Diversity and Environmental Relationships in the Jinsha River During the Initial Phases of the 10‐Year Fishing Ban: A Metabarcoding Approach.” Ecology and Evolution 15, no. 8: e72002. 10.1002/ece3.72002. 40823043 PMC12351800

[ece374041-bib-0095] Zhou, C. , K. Ma , T. Wang , et al. 2025. “Assessing Current Fish Diversity in the Yellow River Basin by Integrating Large‐Scale Barcoding and Morphological Data.” Ecology and Evolution 15, no. 12: e72617. 10.1002/ece3.72617. 41438958 PMC12719916

[ece374041-bib-0096] Zinger, L. , A.‐S. Benoiston , Y. Cuenot , et al. 2025. “Elusive Tropical Forest Canopy Diversity Revealed Through Environmental DNA Contained in Rainwater.” Science Advances 11, no. 33: eadx4909. 10.1126/sciadv.adx4909. 40802774 PMC12346338

[ece374041-bib-0097] Zou, N. , S. Wang , W. Qiu , W. Kong , G. Wang , and S. Wang . 2025. “Environmental RNA as a Transformative Tool for Aquatic Ecosystem Health Assessment: Progress and Challenges.” Ecological Indicators 180: 114328. 10.1016/j.ecolind.2025.114328.

